# SCOTROC 2A: Carboplatin followed by docetaxel or docetaxel–gemcitabine as first-line chemotherapy for ovarian cancer

**DOI:** 10.1038/sj.bjc.6602909

**Published:** 2006-01-10

**Authors:** P A Vasey, R Atkinson, R Osborne, D Parkin, R Symonds, J Paul, L Lewsley, R Coleman, N S Reed, S Kaye, G J S Rustin

**Affiliations:** 1 CR-UK Clinical Trials Unit, Beatson Oncology Centre, Western Infirmary, Dumbarton Road, Glasgow G11 6NT, Scotland, UK; 2 Belfast City Hospital HSS Trust, 51 Lisburn Road, Belfast BT9 7AB, UK; 3 Dorset Cancer Centre, Poole Hospital NHS Trust, Longfleet Road, Poole, Dorset BH15 2JB, UK; 4 Aberdeen Royal Infirmary, Foresterhill, Aberdeen AB25 2ZN, UK; 5 University Hospitals of Leicester NHS Trust, Leicester Royal Infirmary, Infirmary Square, Leicester LE1 5WW, UK; 6 Weston Park Hospital NHS Trust, Whitham Road, Sheffield S10 2SJ, UK; 7 Royal Marsden Hospital, Downs Road, Sutton, Surrey SM2 5PT, UK; 8 Mount Vernon Hospital, Rickmansworth Road, Northwood, Hertfordshire HA6 2RN, UK

**Keywords:** ovarian cancer, docetaxel, carboplatin, gemcitabine, triple-agent therapy, sequential therapy

## Abstract

The feasibility of sequential carboplatin followed by docetaxel-based therapy for untreated ovarian cancer was determined. Patients received four q3w cycles of carboplatin AUC 7, then four q3w cycles of either docetaxel 100 mg m^−2^ (day 1) (arm A); docetaxel 75 mg m^−2^ (day 8) and gemcitabine 1250 mg m^−2^ (days 1,8) (arm B) or docetaxel 25 mg m^−2^ and gemcitabine 800 mg m^−2^ (both given weekly (days 1,8,15)) (arm C). A total of 44 patients were randomised to each treatment arm. None of the arms demonstrated an eight cycle completion rate (70.5/72.7/45.5% in arms A/B/C, respectively), which was statistically greater than 60% (*P*=0.102, *P*=0.056, *P*=0.982) which was our formal feasibility criteria, although only the completion rate in arm C was clearly worse than this level. The overall response rate (ORR) after carboplatin was 65.7% in 70 evaluable patients. In evaluable patients, ORRs after docetaxel-based cycles were: arm A 84.0% (21 out of 25); arm B 77.3% (17 out of 22); arm C 69.6% (16 out of 23). At follow-up (median 30 months), median progression-free survival times were: arm A 15.5 months (95% CI: 10.5–20.6); arm B 18.1 months (95% CI: 15.9–20.3); arm C, 13.7 months (95% CI: 12.8–14.6). Neutropenia was the predominant grade 3–4 haematological toxicity: 77.8/85.7/54.4% in arms A/B/C, respectively. Dyspnoea was markedly increased in both gemcitabine-containing arms (*P*=0.001) but was worse in arm C. Although just failing to rule out eight cycle completion rates less than 60%, within the statistical limitations of these small cohorts, the overall results for arms A and B are encouraging. Larger phase III studies are required to test these combinations.

Primary cytoreductive surgery plus chemotherapy is the standard treatment for patients with advanced epithelial ovarian cancer, with paclitaxel–carboplatin now considered the treatment of choice for first-line chemotherapy (Kaye, 2000). However, despite a modest improvement in survival in recent years, the median 5-year survival for advanced-stage disease remains poor at 31% (American Cancer Society, 2004).

Peripheral neuropathy is a common adverse event associated with paclitaxel-based therapy (Guastalla and Dieras, 2003) and can lead to early treatment discontinuation. Docetaxel is a second-generation taxane that has pharmacological and pharmacokinetic advantages over paclitaxel (Gligorov and Lotz, 2004) and a substantially different toxicity profile to paclitaxel when combined with carboplatin (Hsu *et al*, 2004). Like paclitaxel, docetaxel has significant activity in ovarian cancer (Mäenpää, 2003) and recently the first Scottish Randomised Trial in Ovarian Cancer (SCOTROC 1) established that docetaxel–carboplatin provides similar survival, less neurotoxicity and greater improvement in some quality of life (QoL) parameters compared with paclitaxel–carboplatin (Vasey *et al*, 2004).

The addition of a third cytotoxic agent to a platinum–taxane doublet may potentially improve outcomes in ovarian cancer. High response rates have been reported when gemcitabine is added to a paclitaxel–platinum combination for advanced ovarian cancer (Hansen, 2002), albeit at the expense of significant haematological toxicity (Berkenblit *et al*, 2003; Barlow *et al*, 2004). Likewise, addition of pegylated liposomal doxorubicin (PLD) to frontline carboplatin–paclitaxel as a concurrent triplet necessitates significant PLD dose reduction (Bookman, 2003). Strategies that may potentially reduce such toxicities from triple-agent regimens include sequential scheduling or the use of alternating or sequential doublets. All these are currently being evaluated as an alternative to paclitaxel–carboplatin in the recently completed five-arm trial conducted by the Gynaecologic Oncology Group (GOG) and the International Collaborative Ovarian Neoplasm (ICON) group (GOG-182/ICON5). Survival data are not expected to be reported before 2006.

A sequential-type approach may not only reduce toxicity but might also help to overcome any antagonism which may exist between agents – one possible reason for the failure of paclitaxel–carboplatin to improve survival over carboplatin alone in the ICON3 trial (International Collaborative Ovarian Neoplasm Group, 2002). Further support for a sequential approach is based on studies which show that ovarian cancer cells with abrogated *p53* gene function are sensitised to taxanes (Cassinelli *et al*, 2001), and that ovarian tumours with mutated *p53* are more responsive to taxanes and less responsive to platinum agents than wild-type *p53*-expressing tumours (Lavarino *et al*, 2000; Reles *et al*, 2001). Thus, initial platinum treatment might eradicate one tumour population of wild-type *p53* cells, leaving a population of predominantly mutant *p53* cells amenable to treatment with taxanes. In the clinical setting, results from trial GOG-132 suggest that platinum therapy followed – prior to clinical progression – by paclitaxel does not result in loss of efficacy when compared with concurrent administration (Muggia *et al*, 2000). This randomised study was designed to assess the feasibility of sequentially scheduling single-agent carboplatin followed by docetaxel alone or a docetaxel–gemcitabine doublet as first-line therapy for ovarian cancer.

## PATIENTS AND METHODS

### Patients

Female patients aged ⩾18 years with histologically confirmed epithelial ovarian carcinoma, fallopian tube cancer or ovarian-type primary peritoneal cancer were eligible for the study. Additional inclusion criteria were: International Federation of Gynecologic Oncology (FIGO) stages Ic–IV; Eastern Cooperative Oncology Group performance status ⩽2; no prior chemotherapy or radiotherapy; adequate bone marrow, hepatic and renal function. Exclusion criteria included: mixed mesodermal tumours; tumours considered borderline or ‘possibly malignant’; concurrent malignancy or malignancy within the previous 5 years (except curatively treated uterine cervical carcinoma *in situ* or basal cell skin carcinoma); history of prior serious allergic reactions; pregnancy or lactation; symptomatic peripheral neuropathy ⩾grade 2.

The study had central International Review Board approval and all patients gave written informed consent. Randomisation took place within 8 weeks of initial surgery (laparotomy/biopsy) and patients were allocated to treatment using a minimising algorithm with the following factors: extent of residual disease; centre; FIGO stage; performance status; tumour grade; pretreatment CA-125; presence or absence of primary peritoneal or fallopian tube cancer; interval debulking intention.

### Treatment

Eight cycles (24 weeks) of chemotherapy were planned at 3-weekly intervals. After randomisation, all eligible patients received four cycles of carboplatin AUC (area under the curve) 7 (1-h infusion) on day 1 of each cycle. The dose of carboplatin was derived via the Calvert formula (mg=(glomerular filtration rate+25) × 7) (Calvert *et al*, 1989) and by using ^51^Cr EDTA (edetic acid) measurement of glomerular filtration rate (Chantler *et al*, 1969). This dose remained fixed for all cycles unless toxicity mandated dose reduction.

Patients then received either four cycles of docetaxel 100 mg m^−2^ (1-h infusion) on day 1 (arm A); four cycles of docetaxel 75 mg m^−2^ (1-h infusion) on day 8 plus gemcitabine 1250 mg m^−2^ (30-min infusion) on days 1 and 8 (arm B); four cycles of docetaxel 25 mg m^−2^ plus gemcitabine 800 mg m^−2^, both given as 30-min infusions on days 1, 8 and 15 (arm C). Gemcitabine was always administered after docetaxel.

Premedication for docetaxel comprised dexamethasone 8 mg twice daily for 3 days starting the day before docetaxel administration; oral antiemetics included granisetron 1 mg prechemotherapy and domperidone 20 mg up to four times daily if required.

Cycles were repeated in the absence of progressive disease or prohibitive toxicity. Interval debulking surgery was permitted after finishing carboplatin treatment and before starting docetaxel-based therapy (provided chemotherapy was restarted within 3 weeks).

### Dose/schedule modifications

Treatment was delayed for up to 2 weeks if the neutrophil count was <1.5 × 10^9^ l^−1^ or the platelet count was <100 × 10^9^ l^−1^. Prophylactic antibiotics were recommended for subsequent cycles following grade 4 febrile neutropenia. This was treated by standard protocols involving hospital admission, intravenous antibiotics and (if required) the use of granulocyte-colony-stimulating factors.

The following dose reductions were indicated at first occurrence of neutrophils <1.5 × 10^9^ l^−1^ or platelets <100 × 10^9^ l^−1^ lasting >1 week but <2 weeks, neutropenic fever, or complicated grade 4 thrombocytopenia: carboplatin reduction to AUC 6 (all arms), and a docetaxel reduction to 75 mg m^−2^ in arm A or a 25% reduction for both gemcitabine and docetaxel in arms B and C. At the second occurrence of these haematological toxicities, carboplatin was reduced to AUC 5, docetaxel reduced to 60 mg m^−2^ (arm A), and both drugs reduced by a further 25% (arms B/C). For grade 3–4 nonhaematological toxicity during docetaxel-based cycles, treatment was delayed until toxicity reversed to ⩽grade 1; the doses on subsequent cycles were then reduced by 25%. Treatment delay for ⩽2 weeks was allowed for mucositis grade ⩾3, painful or troublesome mucositis grade 2 and skin toxicity grade ⩾2; subsequent doses were reduced by 25%. In an attempt to improve on the initially poor dose intensity observed in arm C, the protocol was amended in June 2001 to lower the threshold for dose reductions to a neutrophil count of <1.0 × 10^9^ l^−1^ or a platelet count of <75 × 10^9^ l^−1^, with delays for complicated thrombocytopenia mandated until platelets were ⩾75 × 10^9^ l^−1^.

For significant hypersensitivity reactions to docetaxel, the infusion was stopped, symptoms were treated and patients rechallenged within 3 h without further premedication (if appropriate). Milder reactions were managed by slowing the infusion rate, observation until recovery and then reinfusion at the initial rate.

### Clinical assessments

Before study entry, patients underwent a physical examination, abdomino–pelvic computed tomography scan, electrocardiogram, chest X-ray, CA-125, full biochemical profile, full blood count and glomerular filtration rate measurement. Blood counts and biochemical profiles were taken at the start of each carboplatin cycle and weekly during docetaxel-based treatment. CA-125 level was measured at the start of each cycle. Efficacy was assessed using modified Southwest Oncology Group Solid Tumour Response Criteria and by CA-125 (Rustin *et al*, 1996). Quality of life was evaluated before starting treatment and before each treatment cycle using the European Organisation for Research and Treatment of Cancer questionnaires QLQ-C30 and QLQ-OV28 (Cull *et al*, 2001). Toxicities were documented throughout chemotherapy using the National Cancer Institute Expanded Common Toxicity Criteria (NCI-CTC, Version 2.0). Neurotoxicity was assessed using a structured neurological questionnaire and examination before study entry and after four and eight cycles (Cassidy *et al*, 1998).

All patients were followed up every 2 months until progressive disease was documented. Neurological and QoL assessments were continued every 4 months for up to 2 years.

### Statistical considerations

The primary end point for this randomised feasibility study was the percentage of patients completing eight cycles of chemotherapy within each treatment arm. A completion rate of ⩾80% was deemed clearly acceptable, 60–80% was deemed a ‘grey area’ and ⩽60% was deemed clearly unacceptable. The study was designed to test the null hypothesis that the completion rate was ⩽60% against the alternative that it was >60%. The one-sided significance level was set at 5% and the power of the study when the true completion rate was 80% was set at 90%; this required 44 patients to be recruited to each treatment arm. The study was not powered to detect significant differences in efficacy between the three arms; randomisation was utilised in order to ensure that patients with similar characteristics received each of the three options. However, it also permitted a preliminary analysis to be made of the range of efficacy to be expected.

Protocol-defined secondary end points were: toxicities, QoL, clinical response rates and CA-125 responses, and progression-free and overall survival (in particular, at 8 months) (end of treatment). Toxicities and changes in QoL during taxane-based therapy were compared between arms using the Mann–Whitney *U*-test. Quality of life end points were examined in two sets: global health status and functional scales, and symptom scales (including fatigue, nausea and vomiting, pain, abdominal/gastrointestinal symptoms, peripheral neuropathy and hair loss). The statistical significance of the QoL comparisons was assessed by controlling the false discovery rate at 5% (Benjamini and Hochberg, 1995) within each of these sets. Progression-free survival was measured from the date of randomisation to progression or death from any cause (whichever came first). Survival times were also measured from the date of randomisation to death from any cause. Kaplan–Meier methods were used to generate survival curves and estimate survival rates.

## RESULTS

### Patients

Between September 2000 and January 2002, 132 patients were recruited and randomised (44 to each treatment arm) from 11 centres in UK and four centres in Switzerland. The arms were well balanced with respect to baseline demographic and disease characteristics (Table 1).

### Treatment and completion rate

A total of 31 (70.5%; 90% confidence interval (CI): 57.2–81.6%), 32 (72.7%; 90% CI: 59.6–83.4%) and 20 (45.5%; 90% CI: 32.5–58.9%) patients completed the full eight cycles (24 weeks) of planned treatment in arms A, B and C, respectively (primary end point). None of the arms demonstrated a completion rate that was statistically greater than 60% (*P*=0.102, *P*=0.056, *P*=0.982), which was our formal feasibility criteria; the completion rate in arm C is markedly less than this level.

**Figure 1:**
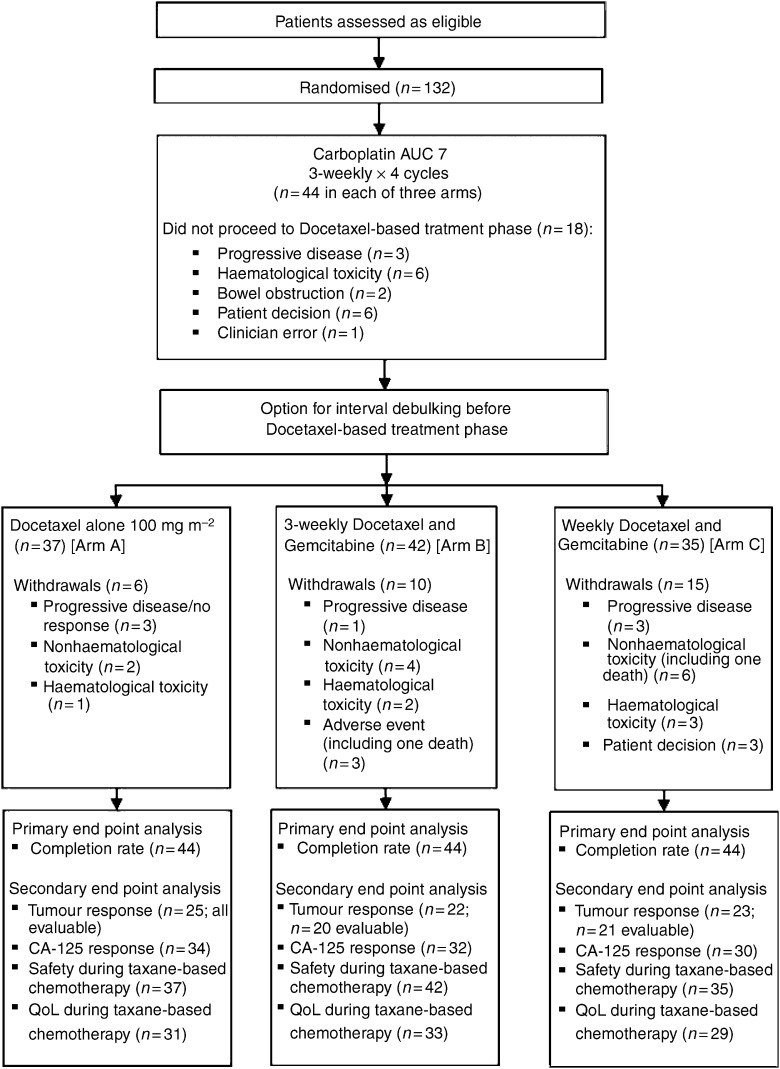
Patient journey through trial.

The progress of patients through the trial is shown in Figure 1. Overall, 121 (91.7%) patients completed their initial four cycles of carboplatin treatment, where 362 out of 505 (71.7%) cycles were delivered on time; 48 (36.4%) patients had no delays, while 41 (31.1%) experienced a delay of one cycle. In most cases (86 (65.2%) patients), the planned dose of carboplatin was given and only 12 (9.1%) patients had two or more dose reductions, resulting in a median dose intensity of 92%.

Of the patients who proceeded to docetaxel-based therapy, 31 out of 37 (83.8%) patients, 33 out of 42 (78.6%) patients and 20 out of 35 (57.1%) patients completed four cycles of treatment in arms A, B and C, respectively (Figure 1). In arm A, 17 (45.9%) patients had no cycle delays, 16 (43.2%) patients had one cycle delay and four (10.8%) patients had two cycle delays. In arm B, nine (21.4%) patients had no cycle delays and 31 (73.8%) had one to four delays either on day 1 or day 8 of treatment. In arm C, only two (5.7%) patients received all cycles on time and 25 (71.4%) experienced one to four delays.

Dose intensities during docetaxel/gemcitabine chemotherapy were calculated over the period following these agents' first cycle; delays starting docetaxel/gemcitabine as a result of prior carboplatin therapy are not included. The median dose intensities were 99% (docetaxel in arm A), 84% (docetaxel in arm B), 86% (gemcitabine in arm B) and 73% (for both gemcitabine and doceaxel in arm C). The protocol amendment resulted in the dose intensity in arm C increasing from 55 to 74% (gemcitabine) and 55 to 73% (docetaxel).

In total, 24 (64.9%) patients in arm A received the full dose at each cycle and 12 (32.4%) patients had one dose reduction. Full dose was given as planned to most patients in arm B, with 30 (71.4%) and 33 (78.6%) patients receiving full doses of docetaxel and gemcitabine, respectively. In this treatment arm, nine (21.4%) patients experienced one docetaxel dose reduction and four (9.5%) had one gemcitabine dose reduction. Dose reductions were more common in arm C, with only 17 (48.6%) patients having no dose reductions and 14 (40.0%) patients having one dose reduction.

### Response

Clinical response data were available for 70 patients with measurable disease during the carboplatin phase of the study, although seven became unevaluable for various nonclinical reasons (e.g. wrong investigation performed). The overall response rate after this phase was 65.7% (46 out of 70), including 14 (20.0%) complete and 32 (45.7%) partial responses. A total of 12 (17.1%) patients had stable disease and five (7.1%) patients had progressive disease. Data on tumour responses at the end of docetaxel-based treatment were available for 25, 22 and 23 patients with measurable disease in arms A, B and C, respectively, although four patients subsequently became unevaluable (Table 2). The overall response rates after all planned chemotherapy were 84% (10 complete and 11 partial responses), 77.3% (10 complete and seven partial responses) and 69.6% (seven complete and nine partial responses) in arms A, B and C, respectively. Of 96 patients evaluable for CA-125 response, 72 (75.0%) responded – most (68 out of 72) before docetaxel-based therapy began. Overall, 62.5% (10 out of 16 patients) in arm A, 100.0% (11 out of 11) in arm B and 87.5% (14 out of 16) in arm C had disease which remained stable or improved after the carboplatin phase with the addition of four cycles of docetaxel-based therapy.

### Survival

At the time of this analysis, 86 patients had progressed or died. A total of 79% of surviving patients had a minimum follow-up of 2 years and the median follow-up was 30 months. The 8-month progression-free survival rate was 77.3% (standard error (SE) 6%) in arm A, 93.1% (SE 4%) in arm B and 76.9% (SE 6%) in arm C; the corresponding median progression-free survival times are 15.5 (95% CI: 10.5–20.6), 18.1 (95% CI: 15.9–20.3) and 13.7 (95% CI: 12.8–14.6) months, respectively.

### Toxicity

Table 3 shows the incidence of grade 3–4 haematological toxicity and neutropenic complications occurring during docetaxel-based therapy. Anaemia was more severe in the gemcitabine-containing arms (*P*=0.002), as was thrombocytopenia (*P*<0.001). Neutropenia was more severe in the docetaxel-alone arm (*P*=0.015). No patients had complicated thrombocytopenia. Table 3 also shows the incidence of grade 2–4 nonhaematological toxicity that occurred during docetaxel-based therapy. Alopecia was markedly less in arm C (*P*=0.001). Dyspnoea was markedly increased in both gemcitabine-containing arms (*P*=0.001), but was worse with the weekly regimen (arm C). Furthermore, arm C was associated with pulmonary infiltrates in three patients and one death due to pulmonary toxicity. There was one further on-study death due to neutropenic sepsis, which also occurred in arm C.

### Quality of life

Quality of life data were available for 31, 33 and 29 patients in arms A, B and C, respectively. Median changes in selected QoL measures during docetaxel-based treatment (average recorded during docetaxel-based therapy minus the value at the end of carboplatin treatment) were compared between the treatment arms. After adjustment for multiple testing, the only statistically significant difference was in nausea and vomiting, which was higher in arm C (median change 5.6; range −17.0 to 50) compared to arms A (0; −100 to 20) and B (0; −44 to 33).

## DISCUSSION

Sequential chemotherapy may potentially maximise the impact of each chemotherapeutic agent while avoiding overlapping toxicities caused by concurrent administration. Following the results of ICON-3 and GOG-132, this represents a logical next step in the evolution of induction chemotherapy for ovarian cancer (Muggia, 2003). Our results suggest that sequential chemotherapy with four cycles of carboplatin AUC 7 followed by a 3-weekly schedule of docetaxel-based chemotherapy is feasible as first-line treatment for patients with ovarian cancer. Carboplatin followed by weekly docetaxel–gemcitabine (arm C) was not well tolerated, and dose intensity was compromised as evidenced by more dose delays, dose reductions and a high rate of discontinuations during the docetaxel-based phase (43%). Furthermore, the weekly docetaxel–gemcitabine arm clearly failed the minimum criterion for feasibility (>60% completion rate for eight cycles). Although the lower end of the 90% CIs for completion rates in the docetaxel-alone and 3-weekly docetaxel–gemcitabine arms was just below 60%, their observed completion rates are sufficiently high to make them worthy of consideration for further clinical testing. Comparisons with toxicity seen in other trials and schedules are fraught with imprecision, but the concurrent triplet combination of carboplatin/paclitaxel/gemcitabine appears to produce significant bone marrow suppression which may make this difficult to deliver consistently in the nontrial setting (reviewed by Hansen, 2002).

Dyspnoea was markedly increased in both gemcitabine-containing arms; however, it was much more clinically significant in the weekly docetaxel–gemcitabine arm, which was also associated with pulmonary infiltration in three patients and one death due to pulmonary toxicity. Lung toxicity has been reported with other weekly taxane–gemcitabine schedules – for example, in the SCOTROC 2C trial, where patients were treated with four cycles of carboplatin followed by weekly paclitaxel–gemcitabine in patients with ovarian cancer (Harries *et al*, 2004). One trial with paclitaxel plus weekly gemcitabine was discontinued as four out of 12 patients with non-small-cell lung cancer experienced dose-limiting pneumonitis (Thomas *et al*, 2000). While both docetaxel and gemcitabine can be associated with pulmonary toxicities, it was the weekly schedule that appeared to be associated with the development of pulmonary toxicity in the present trial. Although this regimen led to one fatality, this toxicity was otherwise reversible (resolving with time and corticosteroid use). Further evaluation of this toxicity should be undertaken, as it could impact on protocol design for weekly combinations of these agents.

Sequential scheduling with a platinum agent followed by a taxane – with or without other chemotherapeutic agents – has been investigated in several early phase studies in ovarian cancer. Unacceptable neurotoxicity with sequential cisplatin (100 mg m^−2^ for four cycles) and paclitaxel (200 mg m^−2^ for four cycles) has been reported (Poole *et al*, 2000), although in most cases sequential scheduling allowed planned dose intensity to be achieved (Tognoni *et al*, 2000; Guppy *et al*, 2004). Importantly, sequential scheduling did not incur loss of efficacy; in these studies, median overall survival ranged from 22 to 30 months (Poole *et al*, 2000; Tognoni *et al*, 2000; Guppy *et al*, 2004). In SCOTROC 2C, the response rate in evaluable patients was 84%, with a median progression-free survival of 19.5 months at a median follow-up of 28 months (Harries *et al*, 2004). In our study, the response rate of 66% after single-agent carboplatin was improved following sequential treatment with docetaxel-based regimens. Carboplatin followed by docetaxel alone (arm A) produced the highest response rate (84%, compared with 77% in arm B and 70% in arm C). However, carboplatin followed by 3-weekly docetaxel–gemcitabine (arm B) was associated with the best-observed survival outcome, with a median progression-free survival time of 18.1 months (95% CI: 15.9–20.3) – compared with 15.5 months (95% CI: 10.5–20.6) for arm A and 13.7 months (95% CI: 12.8–14.6) for arm C.

In conclusion, although just failing our formal feasibility criteria, we believe that, all taken together, the observed data for sequential chemotherapy with four cycles of carboplatin followed by 3-weekly docetaxel-based therapy suggests that this is a realistic option for first-line therapy of ovarian cancer. Preliminary results of the SCOTROC 2B study, which investigated carboplatin followed by docetaxel alone or 3-weekly docetaxel–irinotecan (Jayson *et al*, 2003), further support this sequential approach and suggest further investigation in ovarian cancer is warranted. Following the results of SCOTROC 2C (Harries *et al*, 2004), in which pulmonary toxicity was noted with the weekly paclitaxel–gemcitabine schedule, a further feasibility study with the 3-weekly paclitaxel–gemcitabine schedule has also been initiated. We aim to analyse the results of all the SCOTROC 2 trials together (including docetaxel and paclitaxel-based schedules) in order to evaluate the optimal sequence and drug combination for future Phase III testing of a sequential approach to standard, concurrent two-drug chemotherapy administration.

## Figures and Tables

**Table 1:** Patient baseline characteristics

	**Docetaxel alone (arm A) (*n*=44)**		**Docetaxel–gemcitabine (3-weekly) (arm B) (*n*=44)**		**Docetaxel–gemcitabine (weekly) (arm C) (*n*=44)**	
	**%**	**Count**	**%**	**Count**	**%**	**Count**
*Residual bulk*						
None/micro	36.4	16	34.1	15	36.4	16
⩽2 cm	29.5	13	27.3	12	29.5	13
>2 cm	34.1	15	38.6	17	34.1	15
						
*FIGO stage*						
Ic	4.5	2	6.8	3	6.8	3
II	15.9	7	9.1	4	11.4	5
III	65.9	29	70.5	31	68.2	30
IV	13.6	6	13.6	6	13.6	6
						
*Tumour grade*						
Well	9.1	4	2.3	1	4.5	2
Moderate	25.0	11	25.0	11	27.3	12
Poor	56.8	25	61.4	27	56.8	25
Unknown	9.1	4	11.4	5	11.4	5
						
*ECOG PS*						
0	36.4	16	38.6	17	38.6	17
1	59.1	26	54.5	24	54.5	24
2	4.5	2	6.8	3	6.8	3
						
*Intent to have debulking operation*						
No	81.8	36	84.1	37	81.8	36
Yes	18.2	8	15.9	7	18.2	8
						
*Cancer type*						
Ovarian	90.9	40	90.9	40	90.9	40
Peritoneal	6.8	3	6.8	3	9.1	4
Fallopian	2.3	1	2.3	1	0.0	0
						
*CA-125>ULN prior to chemotherapy*						
No	11.4	5	18.2	8	13.6	6
Yes	88.6	39	81.8	36	86.4	38

CA-125=cancer antigen 125; ECOG PS=Eastern Cooperative Oncology Group Performance Status; FIGO=International Federation of Gynecologic Oncology; ULN=upper limit of normal.

**Table 2:** Tumour responses after eight cycles of chemotherapy (by treatment arm)

	**Carboplatin followed by docetaxel alone (arm A) (*n*=25)**		**Carboplatin followed by docetaxel–gemcitabine (3-weekly) (arm B) (*n*=22)**		**Carboplatin followed by docetaxel–gemcitabine (weekly) (arm C) (*n*=23)**	
**Response**	**%**	**Count**	**%**	**Count**	**%**	**Count**
CR	40.0	10	45.5	10	30.4	7
PR	44.0	11	31.8	7	39.1	9
Stable	8.0	2	9.1	2	8.7	2
Progression	8.0	2	4.5	1	13.0	3
Unevaluable	0.0	0	9.1	2	8.7	2

CR=complete response; PR=partial response.

**Table 3:** Haematological (grade 3–4) and nonhaematological (grade 2–4) toxicities (worst grade over cycles of docetaxel-based chemotherapy received)

		**Docetaxel alone (arm A)**		**Docetaxel–gemcitabine (3-weekly) (arm B)**		**Docetaxel–gemcitabine (weekly) (arm C)**	
**Toxicity**	**Grade**	**%**	**Count**	**%**	**Count**	**%**	**Count**
*Haematological*							
Neutropenia	3	11.1	4	38.1	16	22.9	8
	4	66.7	24	47.6	20	28.6	10
White blood cells	3	40.5	15	40.5	17	42.9	15
	4	24.3	9	7.1	3	5.7	2
Platelets	3	8.1	3	4.8	2	11.4	4
	4	0.0	0	4.8	2	0.0	0
Haemoglobin	3	0.0	0	9.5	4	8.6	3
	4	0.0	0	2.4	1	2.9	1
							
*Nonhaematological*							
Alopecia	2	73.0	27	64.3	27	19.8	7
Nausea	2	13.5	5	9.5	4	28.6	10
	3	0.0	0	7.1	3	2.9	1
Vomiting	2	8.1	3	2.4	1	11.4	4
	3	0.0	0	4.8	2	2.9	1
Sensory neuropathy	2	13.5	5	4.8	2	5.7	2
	3	2.7	1	0.0	0	0.0	0
Motor neuropathy	2	2.7	1	0.0	0	0.0	0
	3	0.0	0	2.4	1	2.9	1
Diarrhoea	2	16.2	6	11.9	5	14.3	5
	3	2.7	1	7.1	3	0.0	0
Constipation	2	16.2	6	9.5	4	11.4	4
	3	0.0	0	2.4	1	5.7	1
Stomatitis	2	29.7	11	21.4	9	2.9	1
	3	5.4	2	0	0	0.0	0
Shortness of breath	2	10.8	4	35.7	15	40.0	14
	3	0.0	0	4.8	2	5.7	2
	4	0.0	0	0.0	0	2.9	1
Abdominal pain or cramping	2	10.8	4	4.8	2	8.6	3
	3	0.0	0	2.4	1	5.7	2
	4	0.0	0	2.4	1	0.0	0
Fatigue (lethargy, malaise, asthenia)	2	43.2	16	48.6	18	51.4	18
	3	5.4	2	2.7	1	17.1	6
